# Supplementary material for: The translation inhibitors kasugamycin, edeine and GE81112 target distinct steps during 30S initiation complex formation
Source: Nat Commun. 2025 Mar 12;16:2470. doi: 10.1038/s41467-025-57731-8 (PMC11903750; doi:10.1038/s41467-025-57731-8)
Supplement: Supplementary file 1 — Supplementary Information [file 41467_2025_57731_MOESM1_ESM.pdf]

# SUPPLEMENTARY INFORMATION
# FOR

## The translation inhibitors kasugamycin, edeine and GE81112 target distinct steps during 30S initiation complex formation

Haaris A. Safdari[1,*], Martino Morici[1,*], Ana Sanchez-Castro[2,*], Andrea Dallapè[1,3], Helge Paternoga[1], Anna Maria Giuliodori[4], Attilio Fabbretti[4], Pohl Milón[2,#], Daniel N. Wilson[1,#]

[1] Institute for Biochemistry and Molecular Biology, University of Hamburg, 20146 Hamburg, Germany.

[2] Laboratory of Biomolecules, Faculty of Health Sciences, Universidad Peruana de Ciencias Aplicadas (UPC), 15023 Lima, Peru.

[3] Department of Cellular, Computational and Integrative Biology – CIBIO, University of Trento, 38122 Trento, Italy.

[4] Laboratory of Genetics of Microorganisms and Microbial Biotechnology, School of Biosciences and Veterinary Medicine, University of Camerino, 62032, Camerino, MC, Italy.

* these authors contributed equally

[#] Correspondence to:

Daniel N. Wilson (Daniel.Wilson@chemie.uni-hamburg.de) and

Pohl Milón (pmilon@upc.pe)

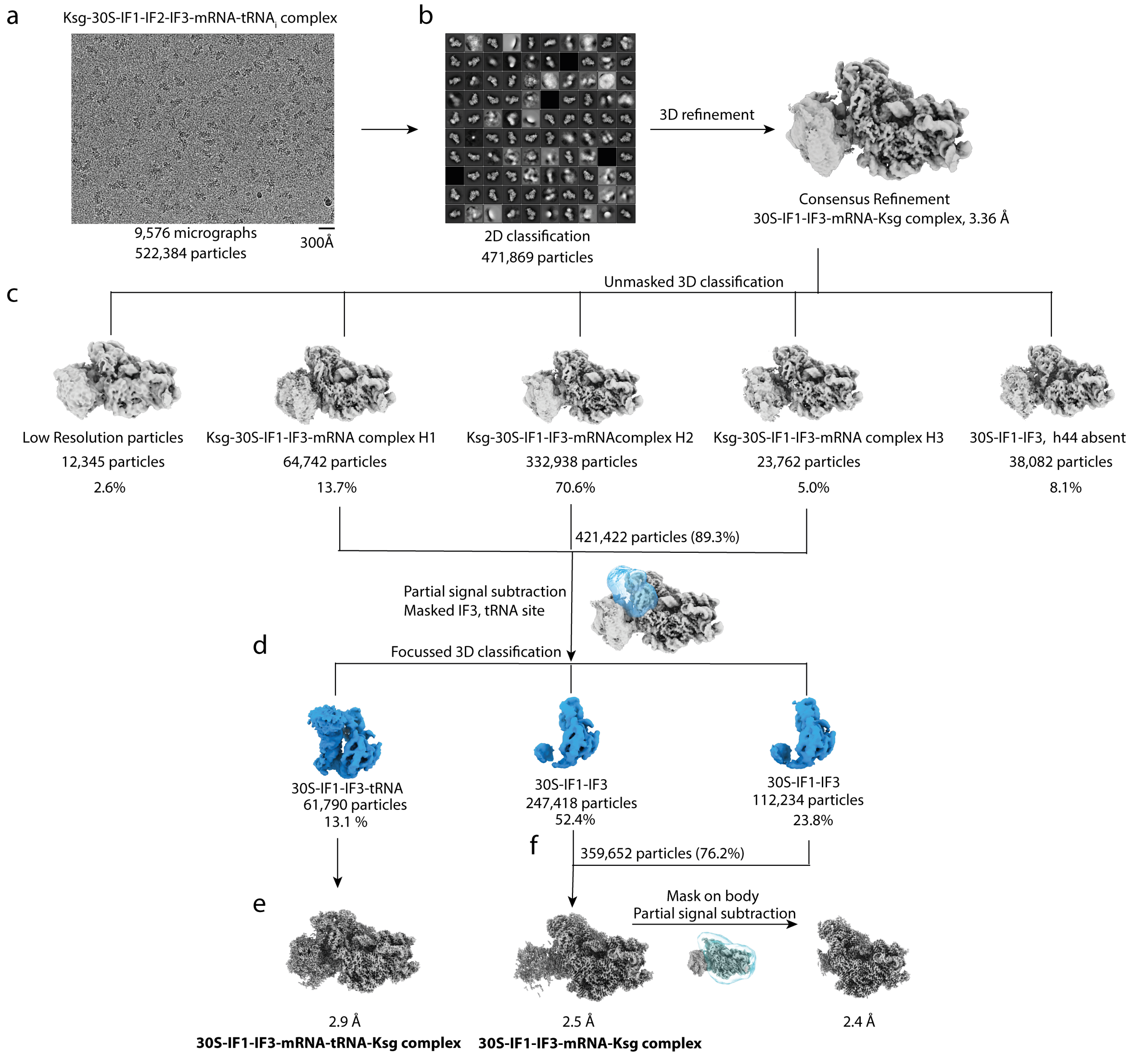


**Supplementary Fig. 1 | *In silico* sorting scheme of *E. coli* Ksg-30S complex. a,** From 9,576 micrographs, a total of 522,384 particles were picked by crYOLO using general model and subjected to 2D classification. **b,** After 2D classification, 471,869 particles were taken for initial consensus refinement. **c,** Unmasked 3D classification into 5 classes was performed. Three classes with Ksg density and different head movement (H1,H2,H3) were merged resulting in 421,422 particles (89.3%). **d,** Another round of focussed 3D classification was performed with a mask surrounding IF3 and tRNA site, yielding three classes, one with density for tRNA (13.1%, 61,790 particles) and other two with no density for tRNA present. **e,** The class with tRNA present (13,1%, 61,790 particles) was refined to high resolution to obtain 2.9 Å resolution. **f,** The latter two classes from (d) without density for tRNA was combined, resulting in 359,652 particles (76.2%). This was further refined to high resolution to reach 2.4 Å resolution. Head was removed from signal subtraction by masking the 30S body since the head was flexible.

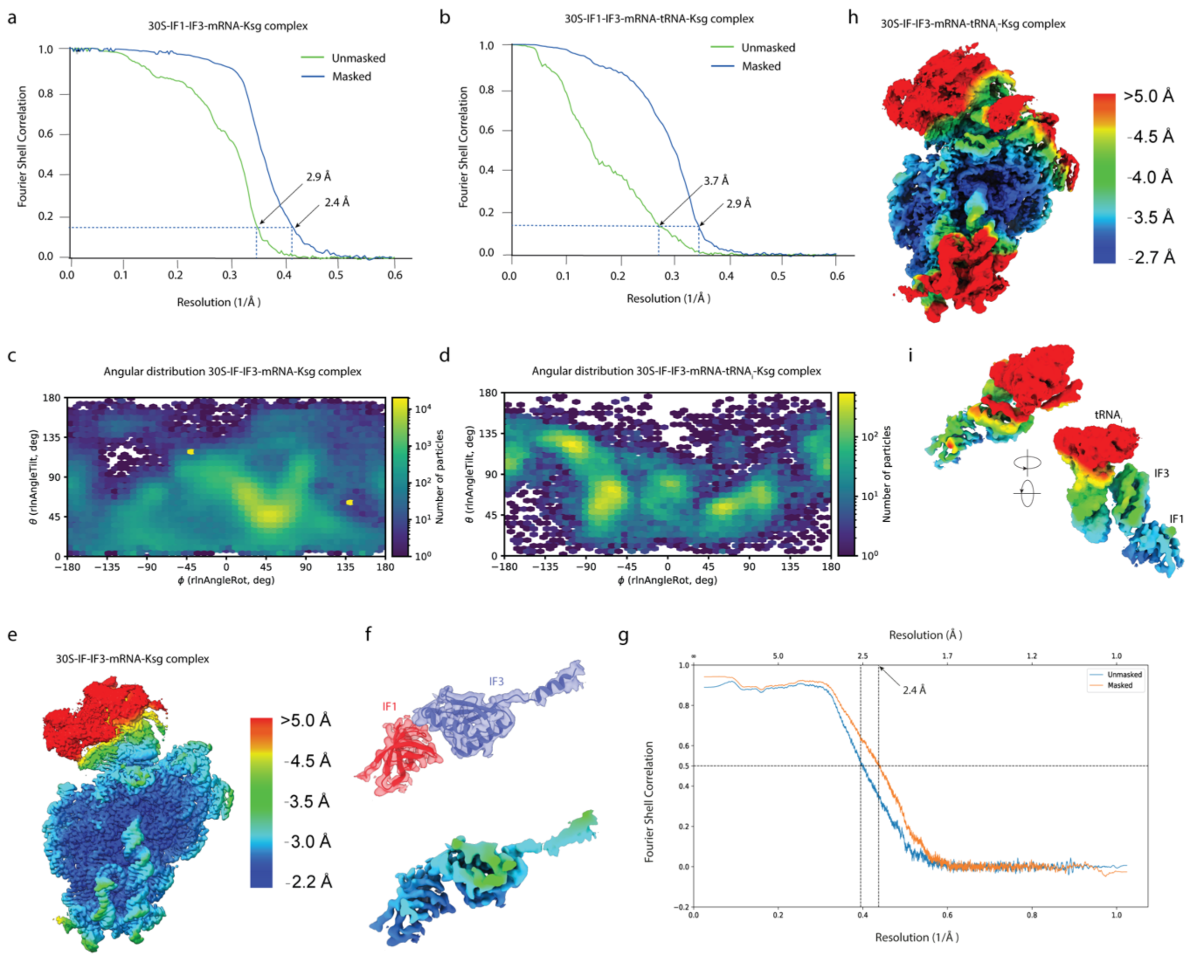

**Supplementary Fig. 2 | FSC and local resolution for *E. coli* Ksg-30S complex. a,** FSC curve for the Ksg-30S-IF1-IF3-mRNA complex map. **b,** FSC curve for the Ksg-30S-IF1-IF3-mRNA-tRNA complex map. **c**, Angular distribution plot for the Ksg-30S-IF1-IF3-mRNA complex map. **d**, Angular distribution plot for the Ksg-30S-IF1-IF3-mRNA-tRNA complex map. **e**, Overview of local resolution of Ksg-30S-IF1-IF3-mRNA complex map. **f**, Isolated densities with fitted models for IF1 and IF3 from map in e, also colored according to local resolution. **g**, FSC map versus model for Ksg-30S-IF1-IF3-mRNA complex map. **h**, Overview of local resolution of Ksg-30S-IF1-IF3-mRNA-tRNA complex map. **i,** Isolated densities of IF1, IF3, tRNA from map in e, colored according to local resolution.

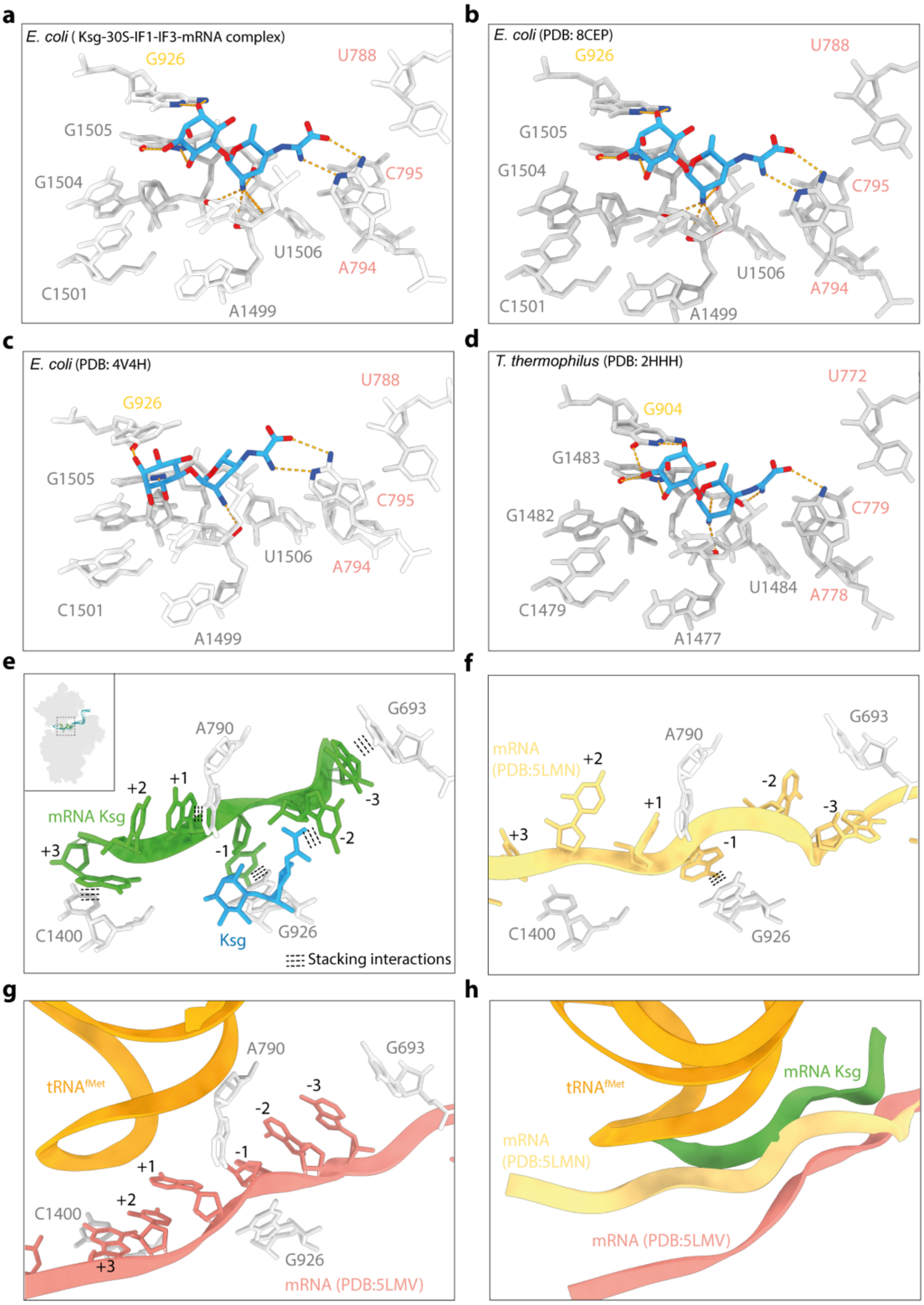


**Supplementary Fig. 3 | Comparison of mRNA path of Ksg and of direct interactions with other structures. a**, Direct interaction of our Ksg structure with *E. coli* 30S ribosomal subunit. **b,** Direct interaction of Ksg structure from PDB: 8CEP with SSU. **c,** Direct interaction of Ksg structure from PDB: 4V4H with SSU. **d,** Direct interaction of Ksg structure from PDB: 2HHH with SSU. **e**, Stacking of rRNA bases on mRNA nucleotides in our Ksg-30S-IF1-IF3-mRNA complex. **f**, mRNA path of PDB:5LMN shown in same context as e. **g**, mRNA path of PDB:5LMN shown in same context as e. **h,** comparison of mRNA path of our Ksg structure, 5LMN and 5LMV shown in context of tRNA position.

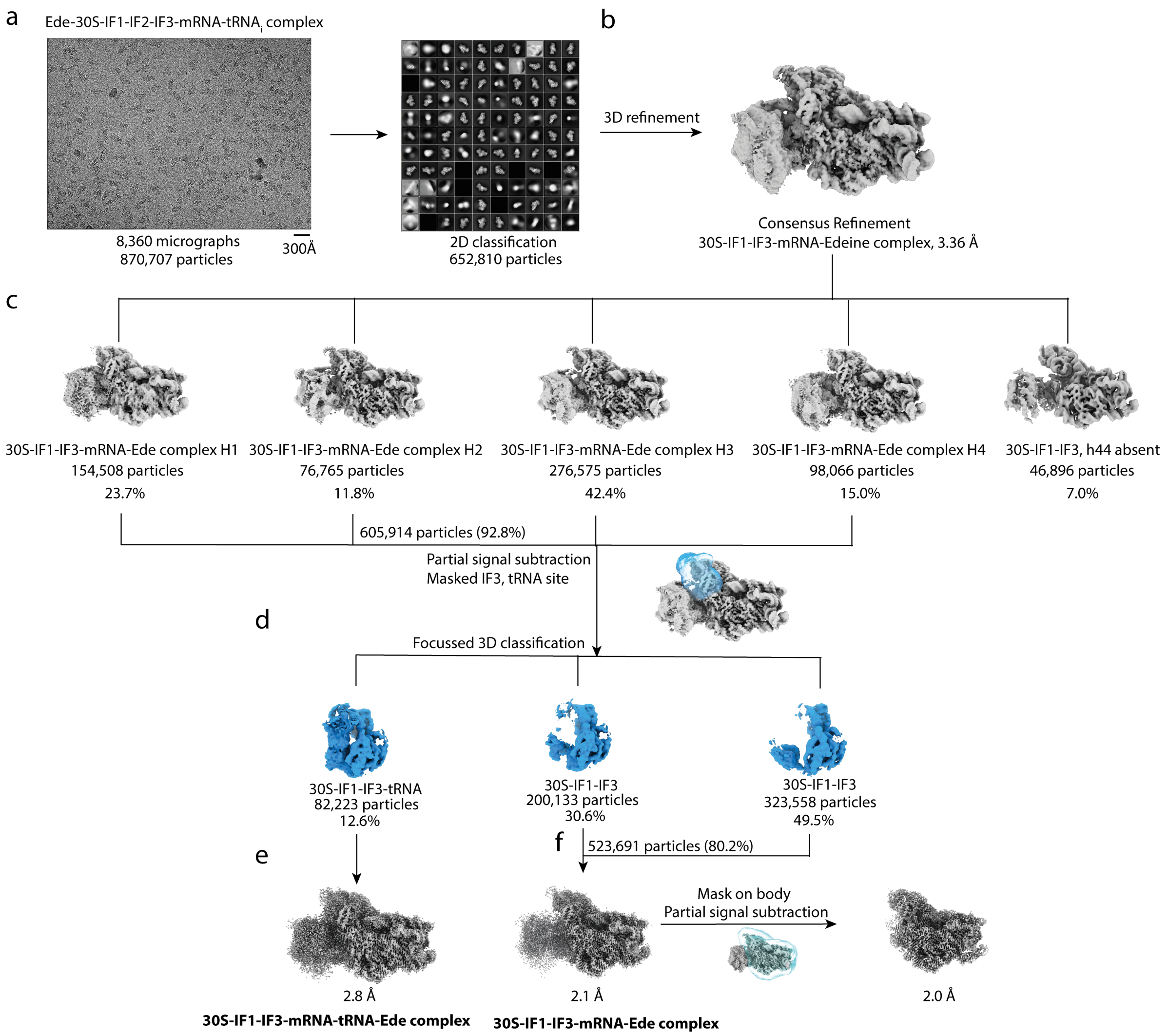


**Supplementary Fig. 4 | *In silico* sorting scheme of *E. coli* Ede-30S complex. a,** From 8,360 micrographs a total of 870,707 particles were picked by crYOLO using general model and subjected to 2D classification. **b,** After 2D classification, 652,810 particles were taken for initial consensus refinement. **c,** Unmasked 3D classification into 5 classes was performed. Four classes with Ede density and different head movement (H1,H2,H3, H4) were merged resulting in 605,914 particles (92.8%). **d,** Another round of focussed 3D classification after signal subtraction was performed with a mask surrounding IF3 and tRNA site, yielding three classes, one with density for tRNA (12.6 %, 82,223 particles) and other two with no density for tRNA present. **e**, The class with tRNA present (12.6%, 82,223 particles) was refined to high resolution to obtain 2.8 Å resolution. **f**, The latter two classed from d without density for tRNA was combined, resulting in 523,691 particles (80.2%). This was further refined to high resolution to reach 2.0 Å resolution. Head was removed from signal subtraction by masking the 30S body since the head was flexible.

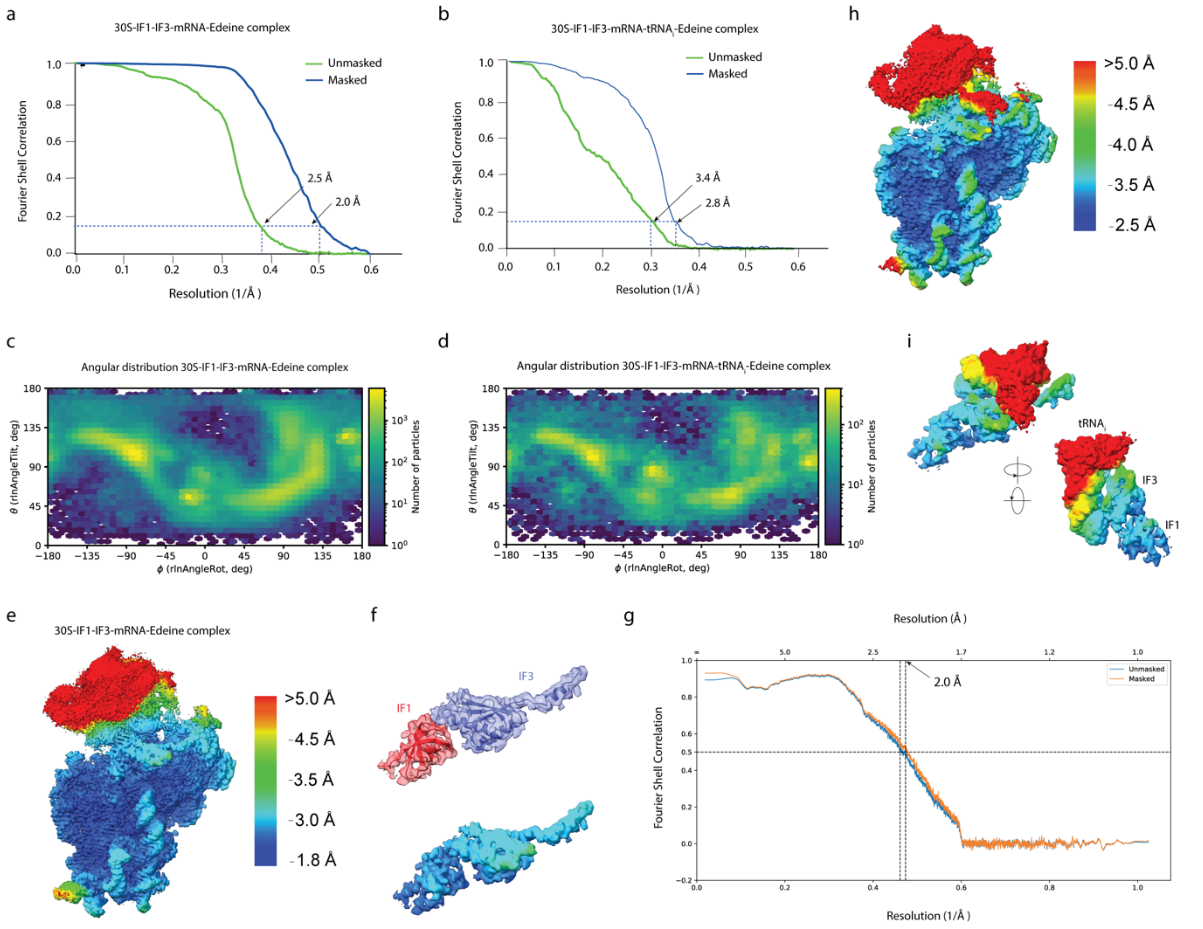


**Supplementary Fig. 5 | FSC and local resolution for *E. coli* Ede-30S complex. a,** FSC curve for the Ede-30S-IF1-IF3-mRNA complex map. **b,** FSC curve for the Ede-30S-IF1-IF3-mRNA-tRNA complex map. **c**, Angular distribution plot for the Ede-30S-IF1-IF3-mRNA complex map. **d**, Angular distribution plot for the Ede-30S-IF1-IF3-mRNA-tRNA complex map. e, Overview of local resolution of Ede-30S-IF1-IF3-mRNA complex map. **f**, Isolated densities with fitted models for IF1 and IF3 from map in e, also colored according to local resolution. **g**, FSC map versus model for Ede-30S-IF1-IF3-mRNA complex map. **h**, Overview of local resolution of Ede-30S-IF1-IF3-mRNA-tRNA complex map. **i,** Isolated densities of IF1, IF3, tRNA from map in e, colored according to local resolution.

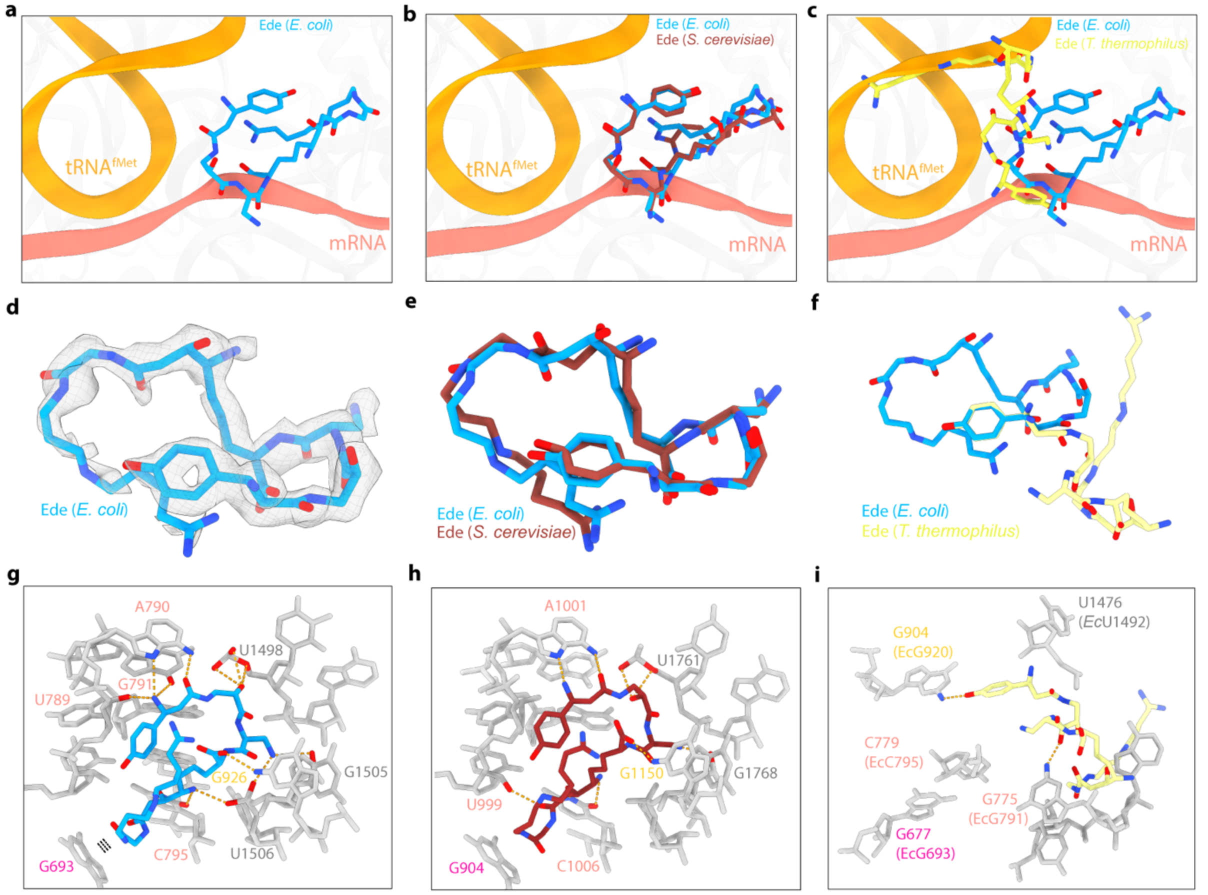


**Supplementary Fig. 6 | Comparison of Ede with other structures. a**, Overview of location of Ede to mRNA and tRNA of 30S-IC (PBD ID 5LMV) as reference. **b,** Overlay of location of our Ede (cyan) in *E. coli* with Ede (brown) from *S. cerevisiae* (PDB ID 4U4N). **c**, Overlay of location of our Ede (cyan) in *E. coli* with Ede (yellow) from *T. thermophilus* (PDB ID 1I95). **d**, Density for Ede from our refined map with lower threshold covering tail of Ede. **e**, Overlay of our Ede (cyan) in *E. coli* with Ede (brown) from *S. cerevisiae* (PDB ID 4U4N). **f**, Overlay of our Ede (cyan) in *E. coli* with Ede (yellow) from *T. thermophilus* (PDB ID 1I95). Alignment done based on isotyrosine ring of respective Ede structures. **g**, Direct interaction of our Ede with *E. coli* SSU. **h**, Direct interaction of Ede (PDB ID 4U4N) on *S. cerevisiae* ribosomes SSU. **i**, Direct interaction of Ede (PDB ID 1I95) on *T. thermophilus* ribosomes SSU.

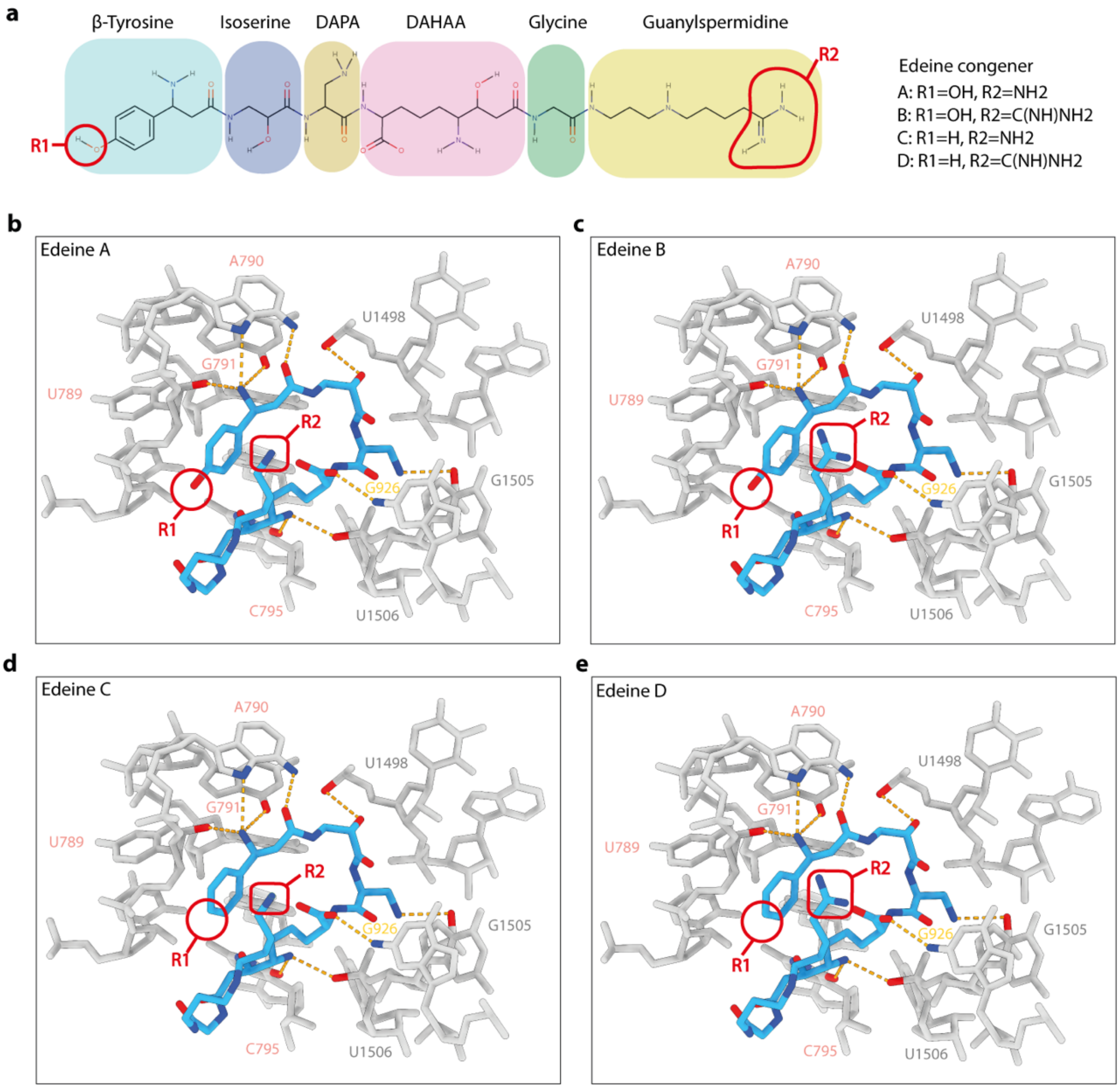


**Supplementary Fig. 7 | Conservation of structures and interactions of Ede congeners on 30S ribosomal subunit. a,** Chemical structure of the Ede B consisting of β-tyrosine, isoserine, DAPA (2,3-diaminopropanoic acid), DAHAA (2,6-diamino-7-hydroxyazelaic acid), and guanylspermidine moities. The region where they differ is highlighted with R1 and R2 with description. Modelled Interaction of 30S ribosomal subunit with R1 and R2 highlighted of (b), Ede A (c) Ede B, (d) Ede C (e) Ede D

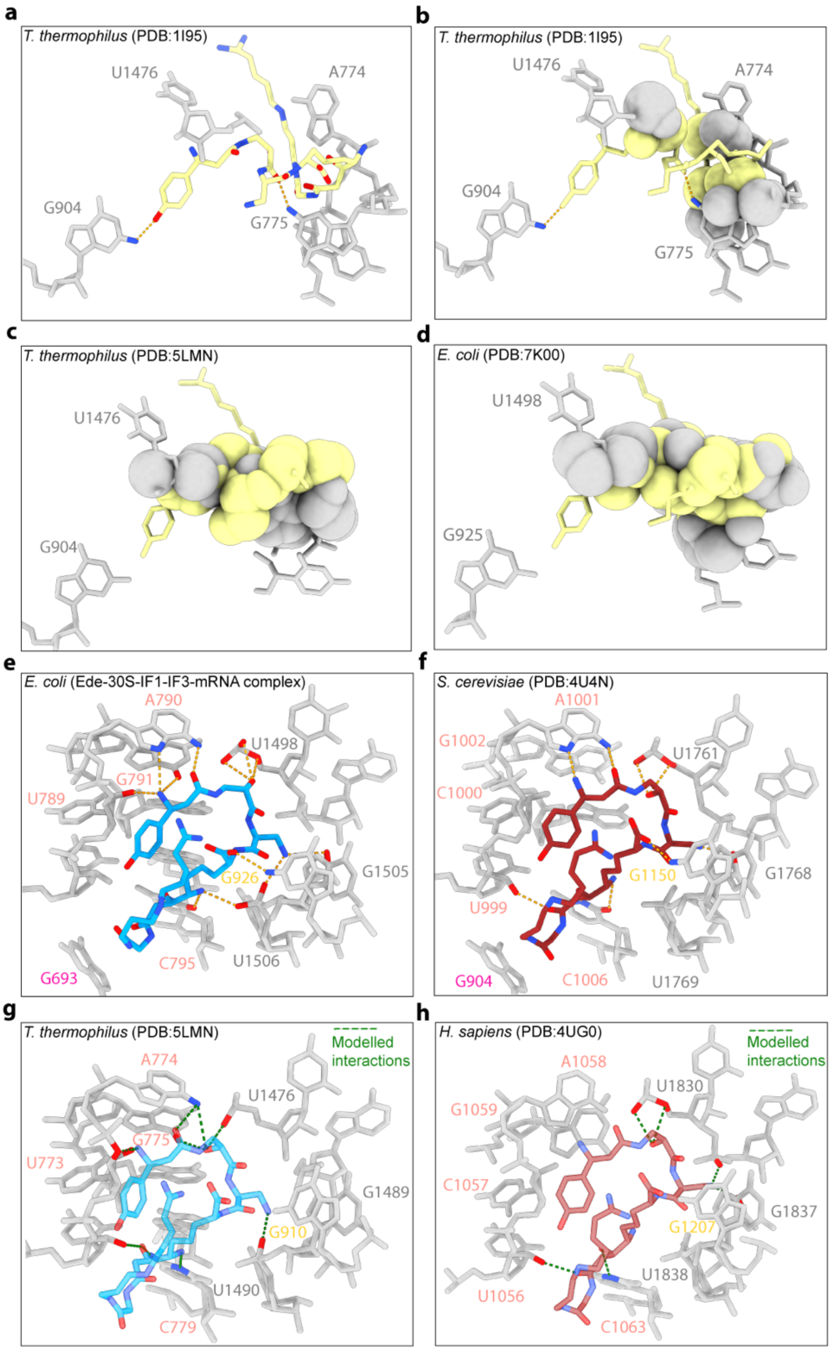


**Supplementary Fig. 8 | Conservation of binding site of Ede in prokaryotes and eukaryotes.** **a**, Direct interaction of Ede (*T. thermophilus*, yellow) from PDB:1I95 with SSU. **b**, Clash of Ede on their own model of *T. thermophilus* ribosomes (PDB ID 1I95) shown as spheres. **c**, Clash of Ede (PDB ID 1I95) on the *T. thermophilus* ribosomes (PDB ID 5LMN) shown as spheres. **d**, Clash of Ede (PDB ID 1I95) on the *E. coli* ribosomes (PDB ID 7K00) shown as spheres. **e**, Direct interaction of Ede (*E. coli*, cyan) with SSU. **f**, Direct interaction of Ede (*S. cerevisiae*, brown) with SSU (PDB:4U4N). **g,** Modelled interaction of our Ede structure on the *T. thermophilus* SSU (PBD ID 5LMN) showing the conservation of binding site in prokaryotes. **h,** Modelled interaction of Ede structure (PDB ID 4U4N) on the *H. sapiens* ribosomes SSU (PDB ID 4UG0) showing the conservation of binding site in eukaryotes.

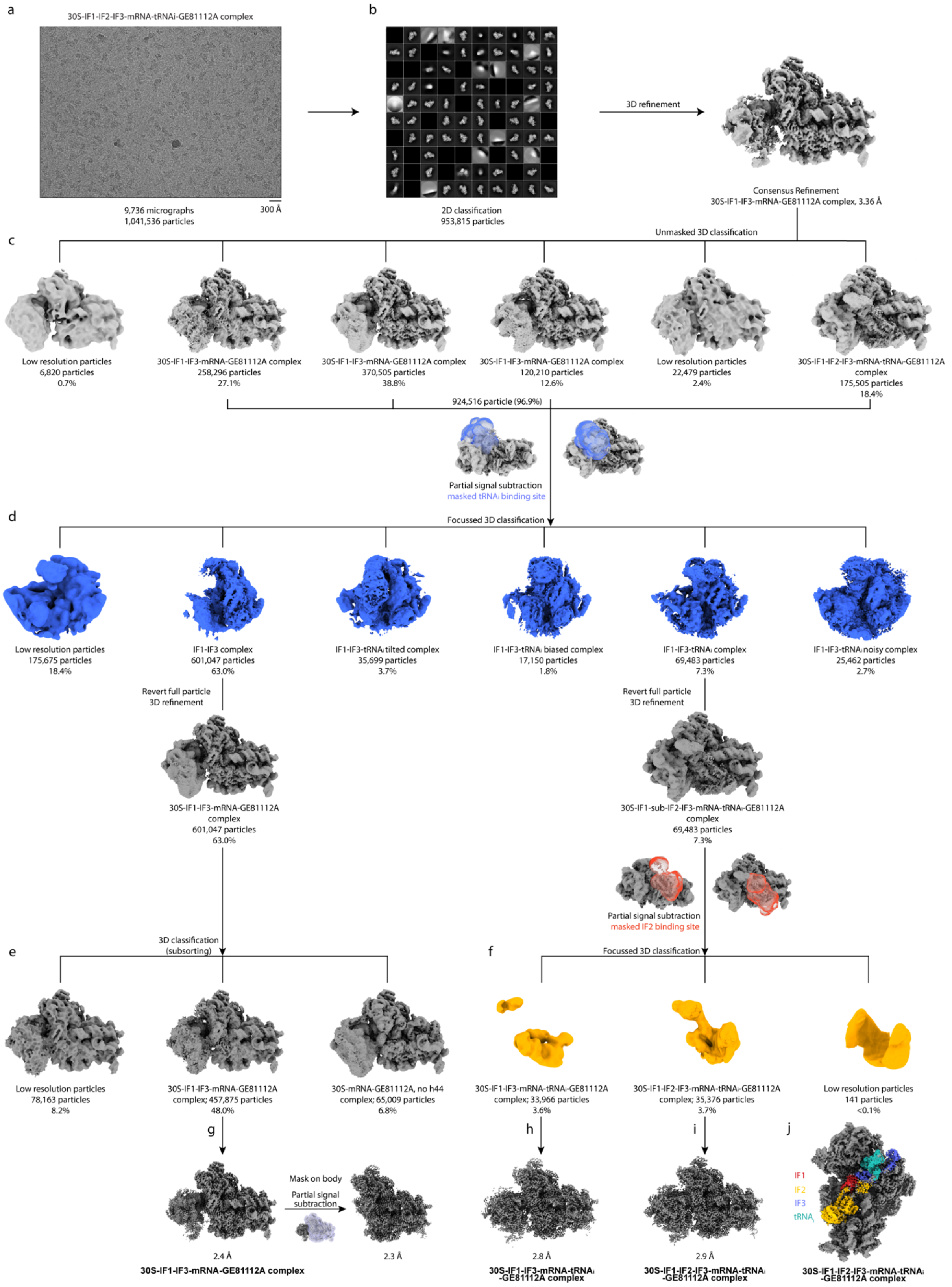


**Supplementary Fig. 9 | *In silico* sorting scheme of *E. coli* GE-30S complex. a,** From 9,736 micrographs, a total of 1,041,536 particles were picked by crYOLO using general model and subjected to 2D classification. **b,** After 2D classification, 953,815 particles were taken for initial consensus refinement. **c,** Unmasked 3D classification into 6 classes was performed. Four high

resolution classes with Ede density were merged resulting in 924,516 particles (96.9%). **d,** Another round of focussed 3D classification after signal subtraction was performed with a mask surrounding tRNA site, yielding six classes, majorly one with density for tRNA (7.3 %, 69,843 particles) and another with no density for tRNA present (601,047 particles, 63%). **e**, The class without tRNA present was subsorted to remove junk particles **f**, Another round of focussed 3D classification was performed on tRNA classes after signal subtraction, with a mask surrounding IF2 site, yielding class with and without IF2. **g**, Class without tRNA was refined to 2.3 Å by masking the 30S body since the head was flexible. **h**, Class with tRNA but no IF2 was further refined to high resolution to reach 2.8 Å resolution. **i**, Class with tRNA and IF2 was further refined to high resolution to reach 2.8 Å resolution. **j**, Map of class with tRNA and IF2 shown at lower threshold.

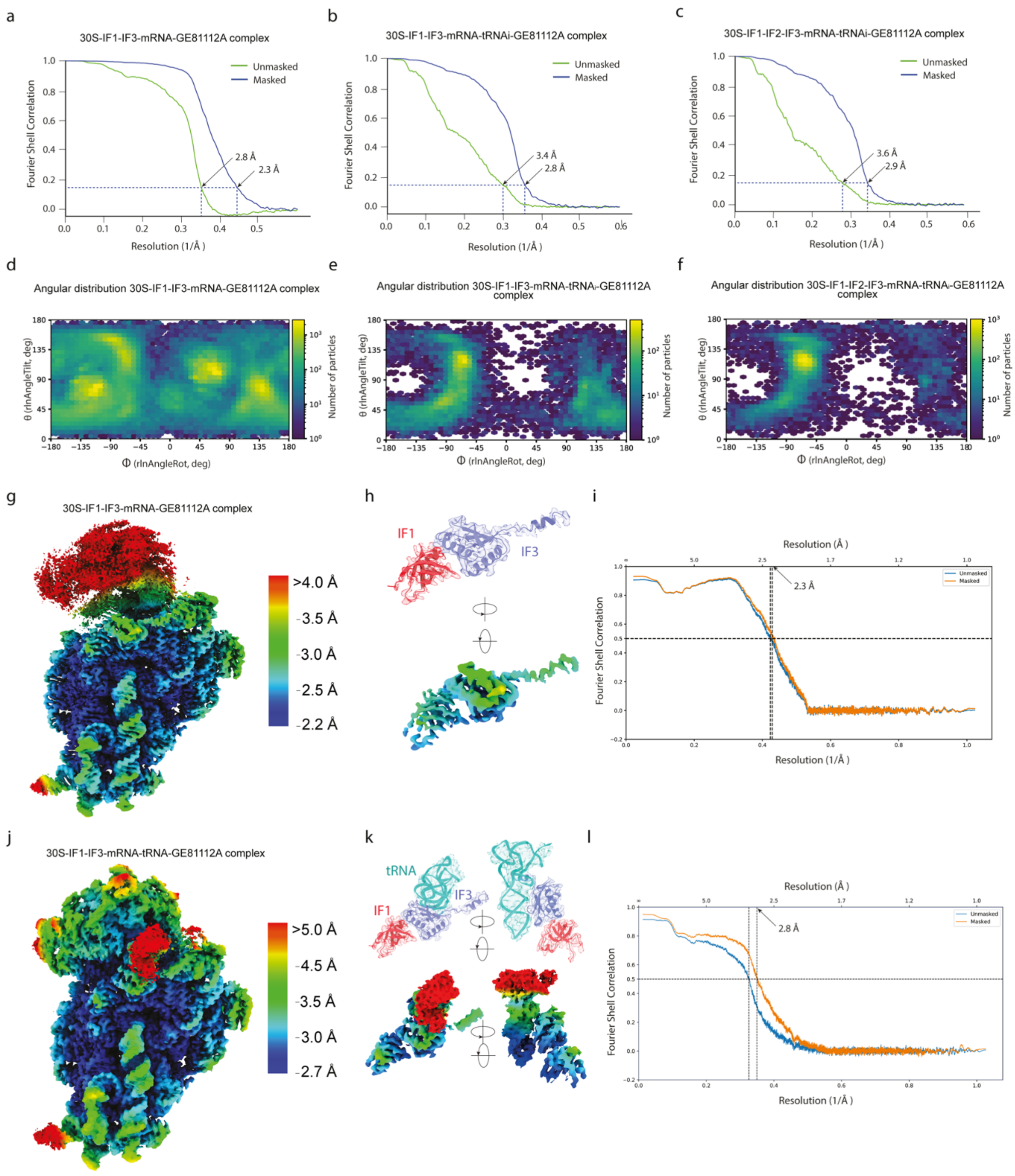


**Supplementary Fig. 10 | FSC and local resolution for *E. coli* GE-30S complex.** FSC curve for (**a**) GE-30S-IF1-IF3-mRNA complex map**,** (**b**) GE-30S-IF1-IF3-mRNA-tRNA complex map, (**c**) GE-30S-IF1-IF2-IF3-mRNA-tRNA complex map. Angular distribution of respective maps in (**d-f**). **g**, Overview of local resolution of GE-30S-IF1-IF3-mRNA complex map. **h**, Isolated densities with fitted models for IF1 and IF3 from map in g, also colored according to local resolution. **i**, FSC map versus model for map in (g). **j**, Overview of local resolution of GE-30S-IF1-IF3-mRNA-tRNA complex map. **k,** Isolated densities with fitted models of IF1, IF3, tRNA from map in (j), colored according to local resolution. **l,** FSC map versus model for map in (j).

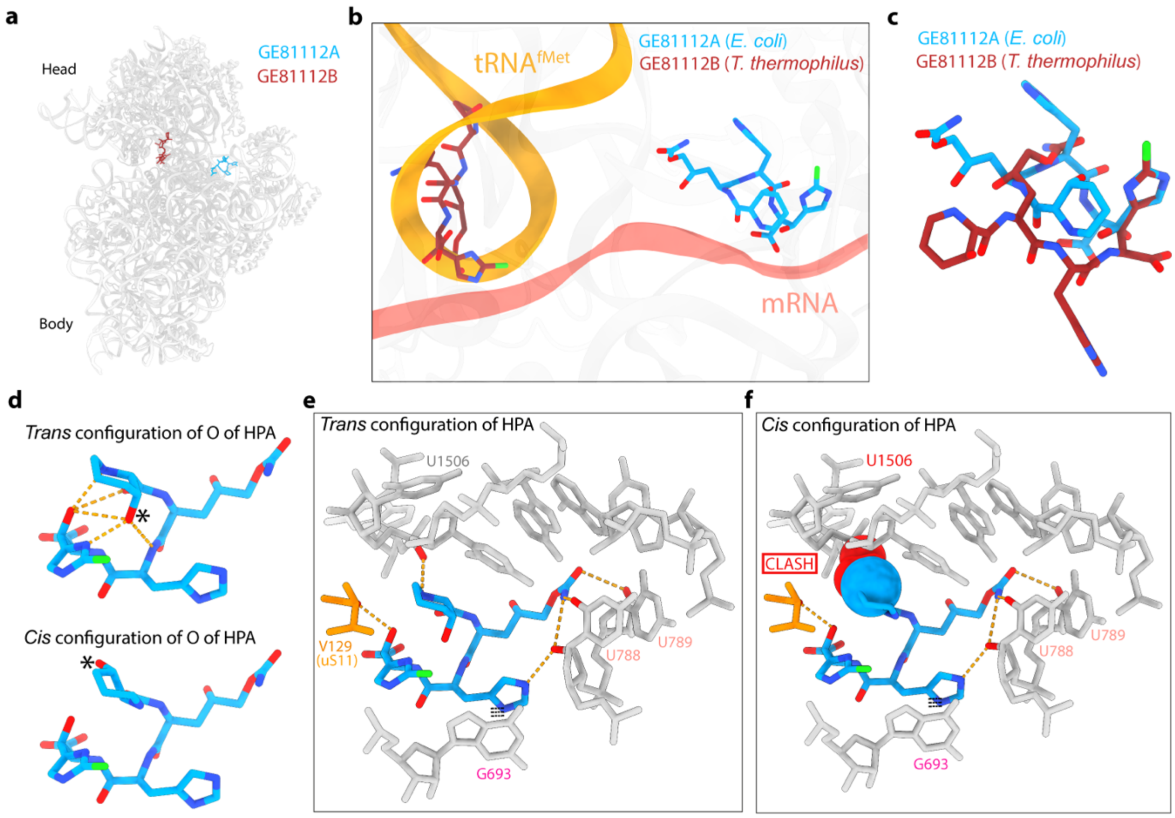


**Supplementary Fig. 11 | Comparison of GE81112A with other structures. a**. Overview of location of GE81112A on 30S in our determined structure with that of GE81112B on *T. thermophilus* ribosome (PDB ID 5IWA). **b**, Overview of location of our GE81112A (cyan) on *E. coli* and GE81112B (brown) on *T. thermophilus* (PDB ID 5IWA) to mRNA and tRNA of 30S-IC (PBD ID 5LMV) as reference. **c**, Alignment of our GE81112A (cyan) in *E. coli* with GE81112 (brown) from *T. thermophilus* (PDB ID 5IWA) based on pentose ring of respective GE81112 structures. **d**, Trans-configuration of oxygen of HPA ring of GE showing several intramolecular H-bonds while *cis* configuration does not form any intramolecular H-bond. Direct interaction of GE with (**e**) trans-configuration, (**f**) *cis* configuration of oxygen of HPA ring of GE.

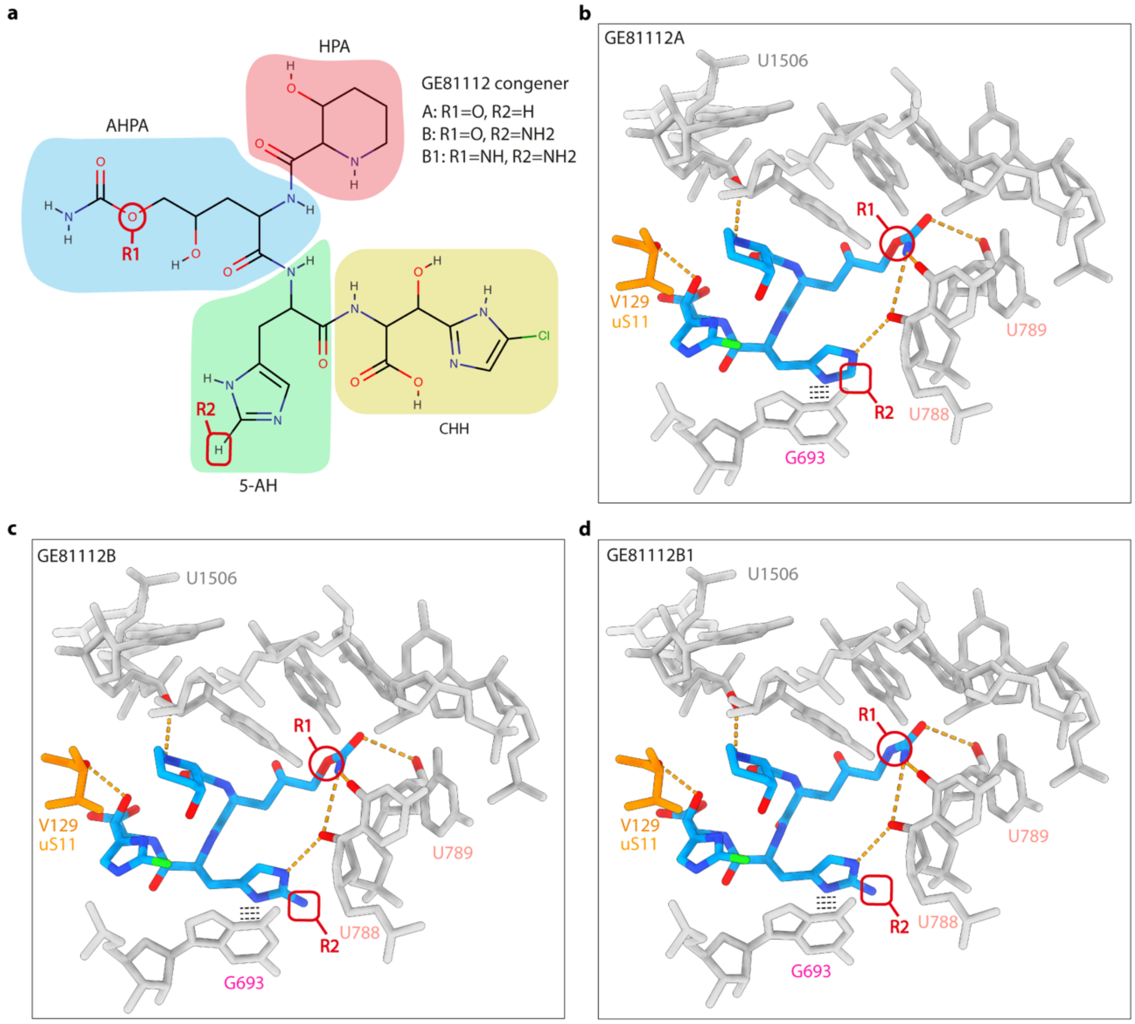


**Supplementary Fig. 12 | Conservation of structures and interactions of GE81112 congeners on 30S ribosomal subunit. a.** Chemical structure of the GE81112A consisting of 5-amino-histidine (5-AH), 3-hydroxypipecolic acid (HPA), 2-amino-5-[(aminocarbonyl)oxy]-4-hydroxypentanoic acid (AAHPA), 5-chloro-2-imidazolylserine (CIS). The region where they differ is highlighted with R1 and R2 with description. Modelled Interaction of 30S ribosomal subunit with R1 and R2 highlighted of (**b**), GE81112A (**c**) GE81112B, (**d**) GE81112B1.

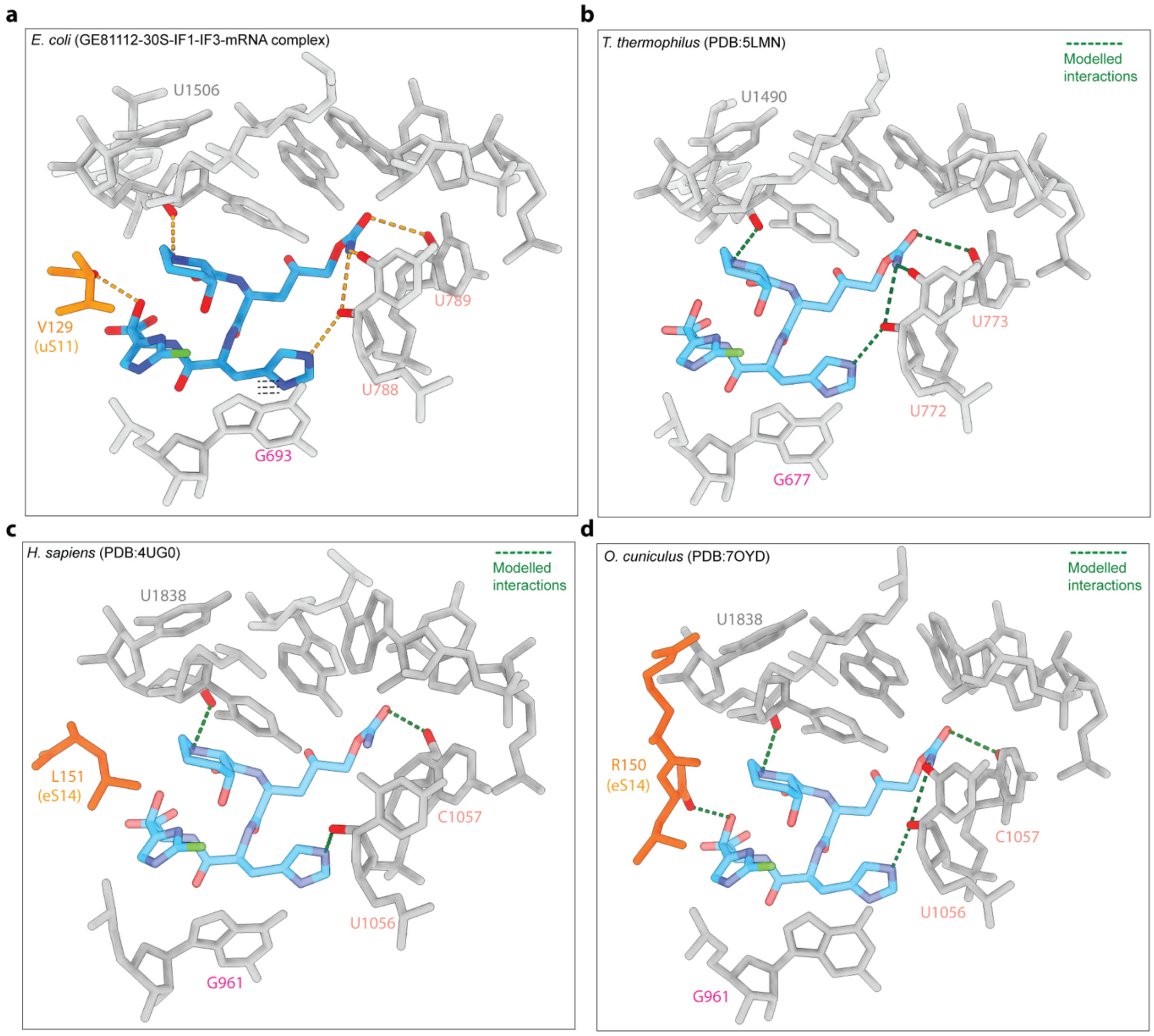


**Supplementary Fig. 13 | Conservation of binding site of GE in prokaryotes and eukaryotes.** **a**, Direct interaction of GE (*E. coli*, cyan) with SSU. **b**, Modelled interaction of our GE structure on the *T. thermophilus* SSU (PBD ID 5LMN) showing the conservation of binding site in prokaryotes. **c**, Modelled interaction of our GE structure on the *H. sapiens* ribosomes SSU (PDB ID 4UG0) and (**d**), on *O. cuniculus* ribosomes (PDB ID 7OYD) showing the conservation of binding site in eukaryotes.

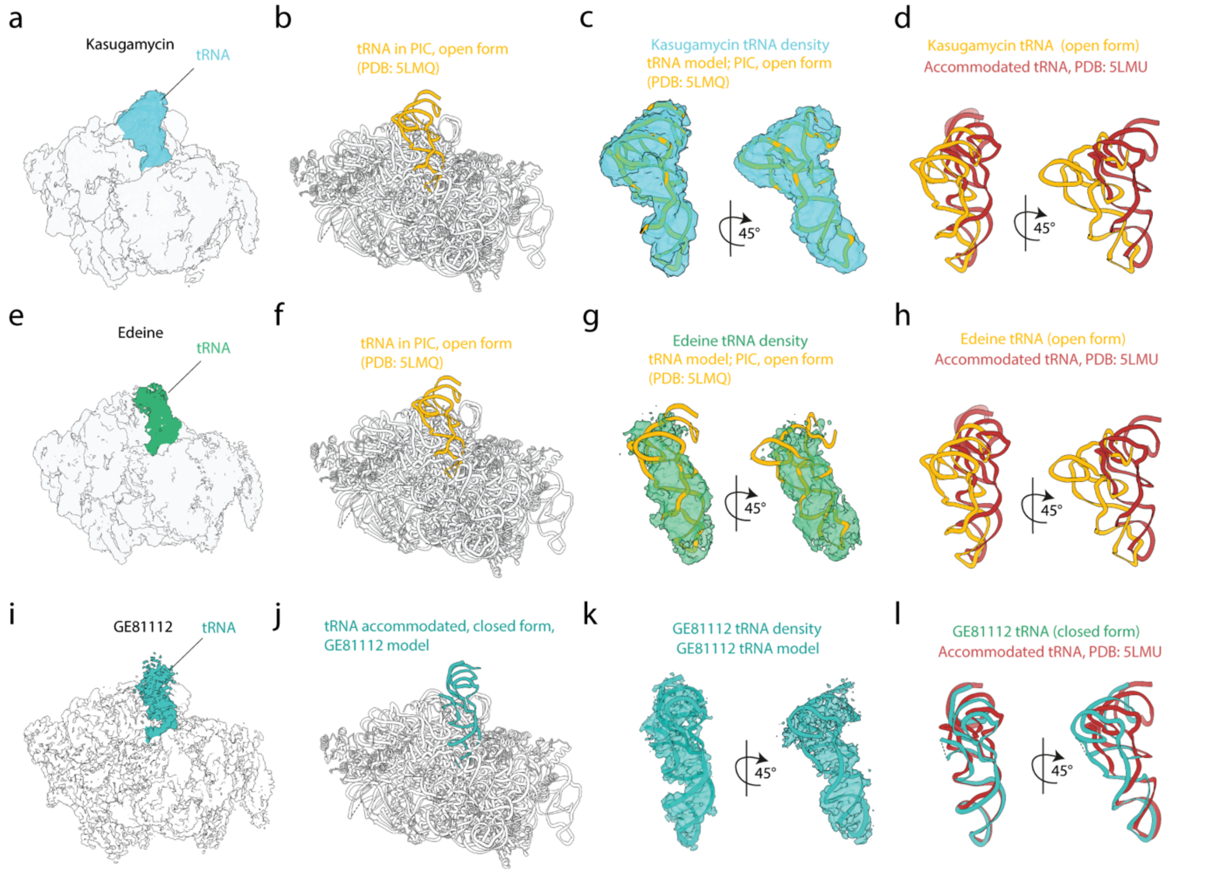


**Supplementary Fig. 14 | Comparison of tRNA conformations from Ksg, Ede, GE structures.** **a,** Side view of Ksg-30S-IF1-IF3-mRNA complex map, with tRNA labelled. **b,** Model of PIC state, open form (PDB:5LMQ) with tRNA highlighted in orange. **c,** Docking of tRNA model from PDB:5LMQ into map in (a) shows a reasonable fit. **d**, Comparison of tRNA conformation that we see in (**a**) (open form), in orange with closed form (accommodated tRNA) from PDB:5LMU in red. **e,** Side view of Ede-30S-IF1-IF3-mRNA complex map, with tRNA labelled. **f,** Model of PIC state, open form (PDB:5LMQ) with tRNA highlighted in orange. **g,** Fitting of tRNA model from PDB:5LMQ into map from (**e**) shows a reasonable fit. **h**, Comparison of tRNA conformation that we see in (**e**) (open form), in orange with closed form (accommodated tRNA) from PDB:5LMU in red. **i**, Side view of GE-30S-IF1-IF3-mRNA complex map, with tRNA labelled. **j,** Model of our complex in (**i**) with tRNA highlighted (light see green). **k,** tRNA density from (**i**) with our model of tRNA **l**, Comparison of tRNA conformation that we see in (**i**) (closed form), in light sea green with closed form (accommodated tRNA) from PDB:5LMU in red.

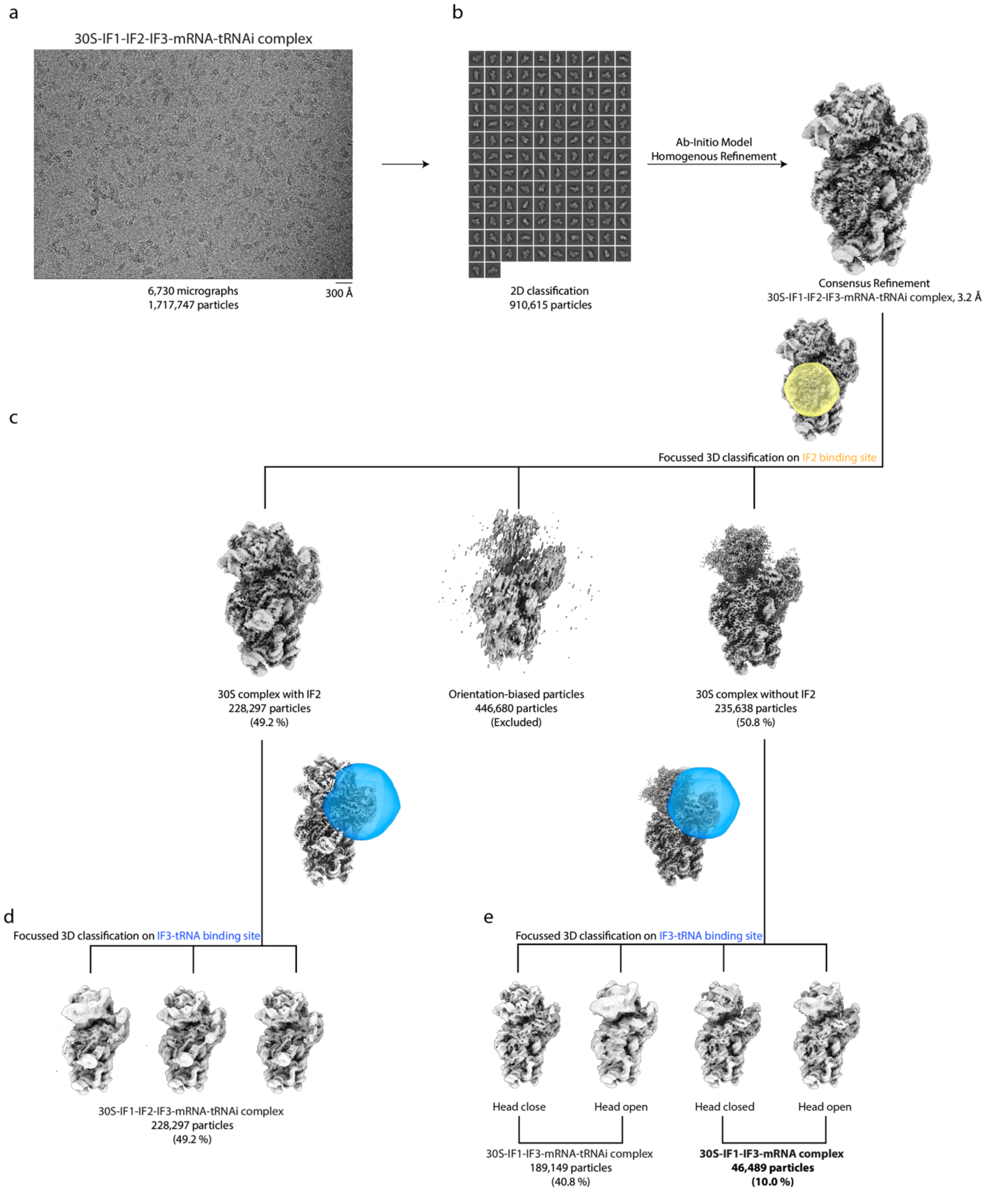


**Supplementary Fig. 15 | *In silico* sorting scheme of *E. coli* 30S initiation complexes in the absence of antibiotic. a,** From 6,730 micrographs, a total of 1,717,747 particles were picked by crYOLO using general model and subjected to 2D classification. **b,** After 2D classification, 910,615 particles were used for ab-initio model and homogenous refinement. **c,** Focussed 3D classification with mask around IF2 into 3 classes was performed. This resulted in class with IF2 (49.2%) and without IF2 (50.8%). Around 446,800 particles were discarded since they showed orientation bias. **d,** Another round of focussed 3D classification with mask around IF3 and tRNA binding site was performed on class with IF2 using a mask surrounding tRNA site, yielding three classes, with minor differences in IF3 conformation. **e**, Similarly, one round of focussed 3D classification with mask around IF3 and tRNA binding site was performed on class

without IF2, yielding four classes. Two of them had tRNA density (189,149 particles, 40.8%) and two others were without tRNA (46,489 particles, 10%).

**Supplementary Table 1 Cryo-EM data collection, refinement and validation statistics**

| | Ksg-PIC (EMDB-50320) (PDB 9FCO) | Ede-PIC (EMDB-50327) (PDB 9FDA) | GE-PIC (EMDB-50476) (PDB 9FIB) | GE-30SIC (EMDB-50912) (PDB 9G06) |
|---|---|---|---|---|
| **Data collection and processing** | | | | |
| Magnification | 105,000x | 105,000x | 105,000x | 105,000x |
| Acceleration voltage (kV) | 300 | 300 | 300 | 300 |
| Electron exposure ($e^{-}$/Å$^2$) | 40 | 40 | 40 | 40 |
| Defocus range (μm) | −0.3 −−1.0 | −0.3 −−1.0 | −0.4 −−1.0 | −0.4 −−1.0 |
| Pixel size (Å) | 0.832 | 0.832 | 0.832 | 0.832 |
| Symmetry imposed | C1 | C1 | C1 | C1 |
| Initial particle images (no.) | 471,869 | 652,810 | 953,815 | 953,815 |
| Final particle images (no.) | 359,652 | 523,691 | 457,875 | 33,966 |
| Map resolution (Å) | 2.4 | 2.0 | 2.3 | 2.8 |
| FSC threshold | 0.143 | 0.143 | 0.143 | 0.143 |
| | | | | |
| **Refinement** | | | | |
| Initial model used (PDB code) | 8CEP | 8CEP | 8CEP | 8CEP,8CA7 |
| Model resolution (masked, Å) | 2.4 | 2.0 | 2.3 | 2.8 |
| FSC threshold | 0.5 | 0.5 | 0.5 | 0.5 |
| CC (mask) | 0.95 | 0.93 | 0.79 | 0.84 |
| CC (volume) | 0.90 | 0.87 | 0.64 | 0.84 |
| Map sharpening *B* factor (Å$^2$) | -32 | -25.7 | -42.3 | -42.45 |
| Model composition | | | | |
| Non-hydrogen atoms | 34,144 | 36,061 | 36,112 | 53,648 |
| Protein residues | 1,445 | 1,457 | 1,443 | 2,257 |
| RNA residues | 1,052 | 1,051 | 1,060 | 1,578 |
| Waters | 1,820 | 1,823 | 1,818 | 1,815 |
| Magnesium (MG) | 52 | 52 | 52 | 52 |
| Potassium (K) | 25 | 25 | 25 | 25 |
| Antibiotics* | KSG | EDE | GE | GE |
| *B* factors (Å$^2$) | | | | |
| Protein | 65.41 | 75.89 | 81.93 | 74.05 |
| RNA | 56.76 | 59.56 | 63.52 | 60.53 |
| Ligand | 58.78 | 56.32 | 57.11 | 57.11 |
| Water | 55.93 | 54.23 | 57.30 | 57.27 |
| R.m.s. deviations | | | | |
| Bond lengths (Å) | 0.009 | 0.009 | 0.008 | 0.010 |
| Bond angles (°) | 1.499 | 1.356 | 1.315 | 1.437 |
| Validation | | | | |
| MolProbity score | 1.09 | 0.99 | 0.89 | 1.25 |
| Clashscore | 1.01 | 0.59 | 0.45 | 1.75 |
| Poor rotamers (%) | 0.58 | 0.66 | 0.25 | 0.32 |
| Ramachandran plot | | | | |
| Favored (%) | 95.75 | 95.72 | 96.45 | 95.46 |
| Allowed (%) | 3.68 | 3.93 | 3.55 | 4.08 |
| Disallowed (%) | 0.57 | 0.35 | 0.00 | 0.45 |
| Ramachandran Z-score | −1.76 | −1.59 | −2.13 | −1.36 |

*KSG (kasugamycin), EDE (edeine), GE (GE81112A)

**Supplementary Table 2 Kinetic parameters for 70S IC formation in the presence of GE, Ede, and Ksg as measured by stopped-flow and light scattering.**

| | $A_{Tot}$ (a.u.) | $k_{fast}$ ($s^{-1}$) | $k_{slow}$ ($s^{-1}$) | $k_{AVG}$ ($s^{-1}$) |
|---|---|---|---|---|
| No antibiotic[1] | 0.07 ± 0.01 | 4.0 ± 1.6 | 0.33 ± 0.07 | 2.0 ± 0.7 |
| GE[1] | 0.04 ± 0.01 | 2.8 ± 2.3 | 0.11 ± 0.01 | 0.7 ± 0.3 |
| Ede[2] | 0.010 ± 0.004 | — | 0.11 ± 0.06 | 0.11 ± 0.06 |
| Ksg[2] | 0.012 ± 0.002 | — | 0.51 ± 0.03 | 0.51 ± 0.03 |

[1]Kinetic parameters were obtained using a two steps exponential function: $A=A1*\exp(-k_{fast}*t)+A2*\exp(-k_{slow}*t)$. [2]Kinetic parameters were obtained using a single step exponential function: $A=A1*\exp(-k_{slow}*t)$. $k_{AVG}=(A1*k_{fast} + A2*k_{slow})/A_{Tot}$. Mean values and standard deviations from three to five replicates are shown.

**Supplementary Table 3: Kinetic parameters for $IF3_{DL}$ dissociation from 30S–IF3 complexes in the presence of GE, Ede, and Ksg as measured by stopped-flow and FRET.**

| | $A_{Tot}$ (V) | $k_{off}$ ($s^{-1}$) |
|---|---|---|
| No antibiotic | 0.58 ± 0.02 | 14.9 ± 3 |
| GE | 0.46 ± 0.02 | 7.2 ± 1 |
| Ede | 0.39 ± 0.03 | 3.5 ± 1 |
| Ksg | 0.66 ± 0.02 | 11.7 ± 2 |

Mean values and standard deviations from three to five replicates are shown.
