# Supplementary material for: The translation inhibitors kasugamycin, edeine and GE81112 target distinct steps during 30S initiation complex formation
Source: Nat Commun. 2025 Mar 12;16:2470. doi: 10.1038/s41467-025-57731-8 (PMC11903750; doi:10.1038/s41467-025-57731-8)
Supplement: Supplementary file 2 — Description of Additional Supplementary Files [file 41467_2025_57731_MOESM2_ESM.pdf]

Description of Additional Supplementary Files

File name: Supplementary Movie 1
Description: Interactions of Ksg with the 30S subunit. Models for Ksg (blue) and 16S rRNA nucleotides (grey) are shown with cryo-EM density. Direct (yellow dashed lines) and indirect (cyan dashed lines mediated by water molecules (red)) interactions are indicated.

File name: Supplementary Movie 2
Description: Interactions of Ede with the 30S subunit. Models for Ede (blue) and 16S rRNA nucleotides (grey) are shown with cryo-EM density. Direct (yellow dashed lines) and indirect (cyan dashed lines mediated by water molecules (red)) interactions are indicated.

File name: Supplementary Movie 3
Description: Interactions of GE with the 30S subunit. Models for GE (blue) and 16S rRNA nucleotides (grey) and uS11 (orange) are shown with cryo-EM density. Direct (yellow dashed lines) and indirect (cyan dashed lines mediated by water molecules (red)) interactions are indicated.

File name: Supplementary Movie 4
Description: Cryo-EM map of GE in the 30S-IC. Overview of the cryo-EM of the GE-30S-IC with 30S (grey), IF1 (bright red), IF3 (blue) and P-tRNA (turquoise). Zoom showing density and models for GE (dark red), P-tRNA (turquoise) and mRNA (green).
